# Characterization and Expression of Sphingosine 1-Phosphate Receptors in Human and Rat Heart

**DOI:** 10.3389/fphar.2017.00312

**Published:** 2017-05-24

**Authors:** Naseer Ahmed, Daniele Linardi, Ilaria Decimo, Riffat Mehboob, Mebratu A. Gebrie, Giulio Innamorati, Giovanni B. Luciani, Giuseppe Faggian, Alessio Rungatscher

**Affiliations:** ^1^Cardiac Surgery Division, University of Verona Medical SchoolVerona, Italy; ^2^Translational Surgery Lab, University of Verona Medical SchoolVerona, Italy; ^3^Section of Pharmacology, Department of Diagnostics and Public Health, University of VeronaVerona, Italy; ^4^Department of Biomedical Sciences, King Edward Medical UniversityLahore, Pakistan

**Keywords:** sphingosine 1-phosphate receptors, heart, human, rat, cardioprotection, ischemia-reperfusion injury

## Abstract

**Aim:** Sphingosine 1-phosphate (S1P), sphingolipid derivatives are known anti-inflammatory, anti-apoptotic, and anti-oxidant agent. S1P have been demonstrated to have a role in the cardiovascular system. The purpose of this study was to understand the precise expression and distribution of S1P receptors (S1PRs) in human and rat cardiovascular tissues to know the significance and possible implementation of our experimental studies in rat models.

**Methods and Results:** In this study, we investigated the localization of S1PRs in human heart samples from cardiac surgery department, University of Verona Hospital and rat samples. Immunohistochemical investigation of paraffin-embedded sections illustrated diffused staining of the myocardial samples from human and rat. The signals of the human heart were similar to those of the rat heart in all chambers of the heart. The immunohistochemical expression levels correlated well with the results of RT-PCR-based analysis and western blotting. We confirmed by all techniques that S1PR1 expressed strongly as compared to S1PR3, and are uniformly distributed in all chambers of the heart with no significant difference in human and rat myocardial tissue. S1PR2 expression was significantly weak while S1PR4 and S1PR5 were not detectable in RT-PCR results in both human and rat heart.

**Conclusion:** These results indicate that experimental studies using S1PR agonists on rat models are more likely to have a potential for translation into clinical studies, and second important information revealed by this study is, S1P receptor agonist can be used for cardioprotection in global ischemia-reperfusion injury.

## Introduction

The S1P is known lysolipid mediator for more than two decades to play a role in cellular changes, including differentiation, proliferation, migration, contraction, and survival (Zhang et al., 1991; Hannun, 1996; Hannun and Obeid, 1997; Spiegel et al., 1998; Ishii et al., 2004; Chun et al., 2010). The lysophospholipid, S1P, is a circulating bioactive lipid metabolite that has been known for many years to induce cellular responses, including proliferation, migration, contraction, and intracellular calcium mobilization (Means and Brown, 2009). There is evidence that S1P can function as an intracellular second messenger (Park Hopson et al., 2011). However, several earlier studies showed that the pertussis toxin that prevents GCPRs response by inhibiting activation of heteromeric G-proteins G_i_ and G_o_ (Mangmool and Kurose, 2011). It can be used to block S1P actions that indicate that S1P works through GCPRs. EDG 1, EDG3, EDG5, EDG6, and EDG8 receptors family of GCPRs showed high affinity for S1P (Hla et al., 2000). It is well-established that the activation of these GCPRs is the mechanism through which most of the biological responses elicit with S1P (Means and Brown, 2009). Recent studies have shown that S1P reduce ischemia-reperfusion injury in liver (Man et al., 2005), kidney (Delbridge et al., 2007), and in the brain (Wacker et al., 2009; Wei et al., 2011). S1P is able to increase the survival of cardiomyocytes during episodes of hypoxia, evidence emerged from *in vitro* studies (Karliner et al., 2001; Tao et al., 2007); it can also reduce the size of the infarcted area in productions of isolated hearts *ex vivo* and *in vivo* model (Jin et al., 2002; Lecour et al., 2002; Santos-Gallego et al., 2016). Although, usually it’s not easy to translate potential therapeutic treatment from animal to clinical settings due to multiple factors mainly including mass specific metabolic rate difference, receptors expression difference in different species (Dobson and Himmelreich, 2002). The aim of this work was to analyze the distribution of S1P receptors in human and rat myocardium and to investigate possible application of rat models for translational studies. According to our knowledge, S1P receptors distribution in the rat is not known yet. We investigated S1PR1, S1PR2, S1PR3, S1PR4, and S1PR5 expression in different chambers of the heart.

## Materials and Methods

This study was conducted in compliance with good clinical practice and according to the Declaration of Helsinki principles. Informed written consent was obtained from all human subjects, under protocols approved by the local Joint Ethical Committee for University of Verona and Hospitals (Verona and Rovigo) for human samples (BBCCH1337). Samples were collected from patients underwent cardiac transplantation in Cardiac Surgery Division, University of Verona Hospital, Verona, Italy. To collect rat samples, healthy Sprague-Dawley male rats weighing 300–350 gm were sacrificed following harvesting of heart that divided into four chambers at C.I.R.S.A.L. (Interdepartmental Research Centre for Laboratory Animals) of the Biological Institutes, University of Verona, and Verona, Italy. The sacrifice of animals was carried out according to the regulations (Declaration of Helsinki and “Guide for the Care and Use of Laboratory Animals” – Institute of Laboratory Animal Resources – National Institutes of Health) after experimental protocols involving animals have been reviewed and approved by the Ethics Committee for University of Verona and the Italian Ministry of Health (341/2016-PR).

Human and rat specimens were collected and fixed in formalin for 24 h, and samples were taken from all the four chambers of the heart. These samples were embedded in paraffin and sectioned into 3-mm-thick slides. Routine staining of hematoxylin-eosin was done to confirm the quality of fixation and paraffin embedding.

### Quantitative Real-Time RT-PCR

The quantitative real-time RT-PCR was performed in Human and rat heart tissue that were collected in liquid nitrogen following storage in -80°C and processed. Total messenger RNA was isolated using a RiboPure (Life Technologies) and quantified by using a Qubit-Fluorometer. The cDNA was extracted containing 1mg of mRNA using a “QuantiTect Reverse Transcription kit” from (Qiagen; Hilden, Germany) RT-PCR was initiated with a denaturation at 93°C for 5 min following 35 cycles at 95°C for next 30 s, at 58°C for another 30 s, and the last extension at 70°C for 12 min. The S1P receptors 1, 2, and 3 specific primers for human and rat were purchased from (Sigma, USA) for each receptor used for performing PCR amplification.

### Western Blotting

Human and rat samples were used for WB. Total proteins were extracted from all the chambers of heart tissue for WB. Myocardial tissues from four chambers were collected separately in cryostat tubes using liquid nitrogen. For protein extraction, RIPA buffer [1% SDS, 1.0 mM sodium orthovanadate, and 10 mM Tris (pH 7.4)] with the addition of protease inhibitor cocktail (Sigma-Aldrich) was used to homogenize. After homogenization, samples were kept for 1 h on ice following 10 min centrifuge at 1600 rpm 4°C temperature to collect supernatant (protein extract). The protein concentration was measured using BCA assays. Then proteins from 20 mg of each of the extracts, were separated by SDS-PAGE 4–12% Bis–Tris gel (Bio-Rad) electrophoresis and transferred to a polyvinylidene difluoride membrane (PDVF), a molecular marker of the Pre-Stained Protein Standard (Sigma-Aldrich) was used as Western blot Protein Standard The PDVF membrane was treated with a 5% FBS at room temperature for 45 min. Primary antibodies were incubated with appropriate dilutions as mentioned in **Table 1**. A mouse anti-GAPDH monoclonal antibody (Sigma-Aldrich; St. Louis, MO, USA) was reacted with the membrane at room temperature for 1 h. The membrane was washed with TBS for 7 min × 3 times and incubated at room temperature for 60 min with a horseradish peroxidase-conjugated anti-mouse/rabbit secondary antibody. The membrane was washed with TBS for 7 min × 3 times, treated with ECL plus Western Blotting Detection System (Syngene box) to develop.

**Table 1:** Primary and secondary antibodies.

Primary antibody	Species type	Clone	Source	Dilution used	Secondary antibody
S1PR1	Rabbit	Polyclonal	Bioss	1:300	Goat anti- rabbit
S1PR2	Rabbit	Polyclonal	John’s Laboratory	1:1000	Goat anti- rabbit
EDG-3	Rabbit	Polyclonal	Bioss	1:300	Goat anti- rabbit
GAPDH	Rabbit	Monoclonal	Cell signaling	1:1000	Goat anti- rabbit

### Immunohistochemistry

Paraffin-embedded samples from human and rat were used for immunohistochemistry. The sections were processed in xylene and serial percentage of ethanol to deparaffinized. Antigen retrieval was carried out in a water bath at 99°C temperature for 30 min and the sections were treated with 3% H_2_O_2_ for 10 min to eliminate endogenous peroxidase activity. After incubation in 5% FBS, the sections were first incubated with the primary antibodies overnight at 4°C temperature. They were then washed in PBS. Then all the sections were incubated in DAB (Dako) for chromogen staining and counterstained with hematoxylin. Negative controls were performed in the same manner but without primary antibodies.

### Statistical Analysis

The RT-PCR data was tested using Student’s *t*-test and Welch’s *t*-test. Statistical significance was considered as a *p* ≤ 0.05.

## Results

**FIGURE 1:**
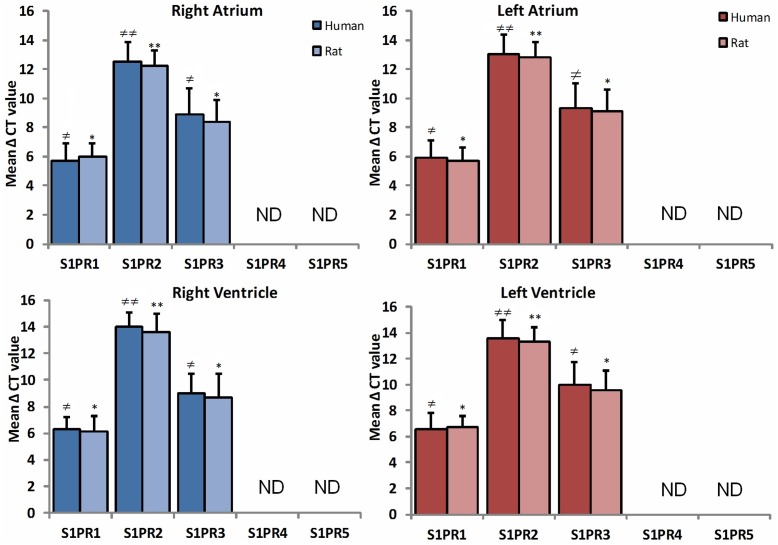
**A comparative study of expression of S1PR between human (*n* = 10) and rat (*n* = 10) heart in all chambers by quantitative real-time RT-PCR.** Data are represented as the mean ΔCt ± SD (ΔCt = Ct value of target mRNA- Ct value of 18S rRNA); therefore, greater expression is equivalent to smaller ΔCt value of mRNA. S1PR1 and S1PR3 mRNA are more abundantly expressed in all chambers of heart as compared to S1PR2. Inversely, S1PR4 and S1PR5 are not detectable in both species (^≠^ *p* ≤ 0.05, ^≠^^≠^ *p* ≤ 0.001 represent human, ^∗^*p* ≤ 0.05, ^∗∗^*p* ≤ 0.001 represent rat).

RT-PCR was done to determine the comparison between human and rat regional distribution of mRNA expression patterns of S1PR1, S1PR2, S1PR3, S1PR4, and S1PR5 (**Figure 1**). The mRNA showed strong expression of S1PR1 in all chambers including right atrium, left atrium, right ventricle, and left ventricle in human and rat both. Mild enhanced expression of S1PR1 was observed in the human heart with statistically no difference in any heart chamber and expression were similar in both human and rat myocardial tissue mRNA. The mRNA level of SIPR2 and S1PR3 were found uniformly distributed in all chambers of human and rat heart. S1PR2 were significantly weak as compared to S1PR1 and S1PR3 (*p* ≤ 0.05). S1PR4 and S1PR5 were not detected either in human or rat heart tissue.

**FIGURE 2:**
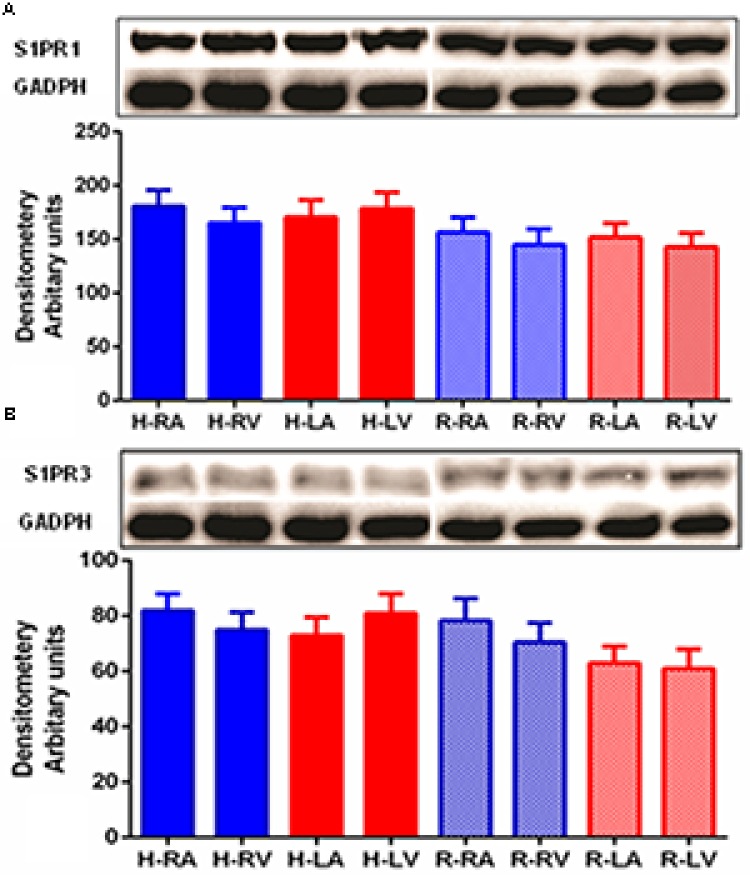
**Detection of S1PR1 (A)** and S1PR3 **(B)** proteins in human and heart by Western blotting. Analysis of S1PR1 and S1PR3 was performed using 50 μg of total proteins of human and rat heart from all four chambers. For S1PR1 detection, the membrane was developed for only 1 min using an ECL(+) kit (Amersham-Pharmacia Biotech), whereas 45 min was necessary for S1PR3. S1PR1 was significantly high as compared to S1PR3 (*p* ≤ 0.05, S1PR1 versus S1PR3 in both species and all chambers). S1PR1 and S1PR3 were uniformly distributed in all part of heart in both species.

Western blot was performed in order to detect S1PR1 and S1PR3 proteins in human and rat heart (**Figures 2A,B**). The S1PR1 and S1PR3 proteins were detected between molecular weight 40–45 kD in myocardial tissues of both species in all chambers. S1PR3 level of expression was relatively lower than S1PR1. Consistently, its distribution was uniformly present in all chambers. Similarly, like S1PR1, S1PR3 was also expressed mildly higher in human samples as compared to rat samples.

**FIGURE 3:**
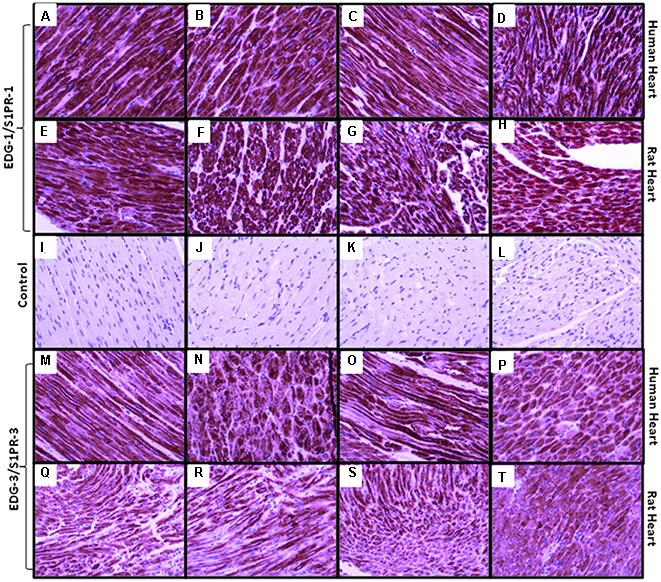
**Representative images of S1PR1 and S1PR3 expression in human and rat heart in right atrium, left atrium, right ventricle and left ventricle** (**A**, **M** = human right atrium, **E**, **Q** = rat right atrium), (**B**, **N** = human right ventricle, **F**, **R** = rat right ventricle), (**C**, **O** = human left atrium, **G**, **S** = rat left atrium), (**D**, **P** = human left ventricle, **H**, **T** = rat left ventricle), (**I–L** are control samples treated same except primary antibody incubation. S1PR1 expressed in all chambers of both species similar, while S1PR3 expressed mildly less in rat samples but also uniform distribution in all chambers). Magnification 20x.

Immunohistochemical staining was performed by anti-S1P1 and anti-EDG-3 (S1PR3) commercially available antibodies for research purpose to localize and analyze the level of expression, in different regions of human and rat heart tissue (**Figure 3**). In immunostaining, perinuclear staining was higher for S1PR1 due to the high density of receptors present. In all experiments, we found a similar pattern of receptor expression in human and rat tissue in all different chambers and non-specific signals were ruled out by using control slides without primary antibody. In immunostaining for S1PR3, uniform distribution of receptors in all chambers was found in both human and rat, rather some difference between human and rat was observed where signals were relatively lower in rats as compared to a human.

## Discussion

During last two decades, ∼1000 experimental studies have been investigated for the cardioprotective role of several drugs (Heusch, 2015). The majority of the preclinical strategies could not work in clinical settings. To minimize this failure, we investigated and compared the S1P receptor expression in human and rat myocardial tissue. Our study, for the first time, provides extensive translational analysis of expression and distribution of S1P receptors at the protein and m RNA level.

First, western blots from rat subjects revealed that EDG1, EDG3, and EDG5 receptors are distributed in all chambers of the heart. They are expressed in all anatomical parts of the adult heart, i.e., right ventricle, left ventricle, right atrium, and left atrium. The S1PR1 mRNA observed throughout heart with the relative high impression in human as compared to rat but that can be due to the affinity of primers. But interestingly, the distribution was similar in all the chambers in the same species that gives a positive way to translate. Consistently, S1PR2 and S1PR3 were distributed uniformly in all chambers. S1PR4 and S1PR5 were not detected as reported by previous authors (Ishii et al., 2001; Mazurais et al., 2002). These results are confirmed at the protein level by Western blotting experiments, where we found a similar pattern in both species and in both species uniform distribution of receptors were observed. However, it is difficult to compare the respective protein amounts because each antibody has a specific affinity and different times of exposure for detection. Taken together, western blotting data provide new insight into previous reports indicating expression of EDG1, EDG3, and EDG5 transcripts in the human (Mazurais et al., 2002) and mouse heart (Zhang et al., 1999).

With the aim of investigating the respective roles played by S1PR1 and S1PR3 in human and heart tissues, it was important to determine the nature of the cells that express the respective messengers or proteins. For the further qualitative study, immunohistochemistry was performed to detect S1PR expression in human and rat heart tissue. It was important to clarify S1PRs localization in human heart because interspecies differences were observed in the EDG-1 receptor (Lado et al., 1994; Liu and Hla, 1997; Zhang et al., 1999). Increased expression of S1PR1 in immunohistochemistry was consistent with the results of mRNA and Western blot for all chambers of the heart as compared to S1PR3. Multiple studies have been done for neonatal cardiomyocytes analysis for S1PR1 and high mRNA expression has been found in atrial and ventricular myocytes (Himmel et al., 2000; Robert et al., 2001).

The pattern of relatively strong signals of S1PR3 compared to S1P2 in all chambers of the heart was similar in human and rat. This finding has been reported previously by Tamama et al. (2000) that S1PR2 in the heart was undetectable or very weakly expressed. S1PR1 and S1PR3 play an important role in the regulation of Akt/erk and connexin43 molecular pathways (Shimizu et al., 2012). These pathways are vital in cardioprotection by reducing apoptosis and inflammation (Bajwa et al., 2010). S1PR1 contribute lymphopenia and mediate cardioprotective effect (Bajwa et al., 2010). S1PR1 agonist has been reported as a protective agent in renal ischemia-reperfusion injury (Bajwa et al., 2010). Multiple studies demonstrated a protective effect in ischemia-reperfusion injury using fingolimod (agonist for S1PR1, S1PR3, S1PR4, and S1PR5) in different organs including the heart (Santos-Gallego et al., 2016). In the light of the above evidence, we suggest that S1PR1 and S1PR3 may play the main role in protection against ischemia-reperfusion. This study suggest new direction that fingolimod can be used for experimental studies for regional as well as global myocardial ischemia-reperfusion injury due to its uniform distribution. To study the effect of S1P, rat models can be best for translation. Although, receptor expression is not the only reason of failure in the translation of preclinical studies to human but it can be a useful tool for this purpose. From this study, it can be assumed that lower dose of S1P can be cardioprotective due to increased expression of S1P receptors in human.

### Limitations

In this study, human samples were taken from transplanted patients with end-stage heart failure versus healthy rats. It needs to rule out in future studies rather heart failure and other cardiomyopathies up or down regulate receptor expression. This study only demonstrates a comparison of receptors expression in different chambers in human and rat heart but need to perform a separate study on cellular level receptor expression in both species. Alone this study is not sufficient to confirm the similar effect of S1P on rat and human, because drug metabolism (Dobson and Himmelreich, 2002) and increased collateral circulation (Schaper, 2009) may cause a difference in human.

## Conclusion

This translational study provides new insights about the expression of S1P receptors in human and rat heart. This study indicates a uniform distribution of S1P1 and 3 in all chambers of the heart in human and rat. These results are suggestive about the significance of rat experimental models to translate into clinical practice.

## Author Contributions

Participated in research design: NA, AR, GF, GL. Conducted experiments: NA, DL, MG. Contributed new reagents: AR, GI. Performed data analysis: NA, RM. Writing manuscript: NA, AR, ID.

## Conflict of Interest Statement

The authors declare that the research was conducted in the absence of any commercial or financial relationships that could be construed as a potential conflict of interest. The reviewer MDB and handling Editor declared their shared affiliation, and the handling Editor states that the process nevertheless met the standards of a fair and objective review.

## Abbreviations

EDG: endothelial differentiation gene
GCPRs: G-coupled protein receptors
S1PRs: sphingosine 1-phosphate receptors
